# Health-Related Quality of Life after Cystectomy and Urinary Diversion for Bladder Cancer

**DOI:** 10.1155/2011/715892

**Published:** 2011-08-07

**Authors:** Cheryl Shih, Michael P. Porter

**Affiliations:** ^1^Department of Urology, University of Washington, Seattle, WA 98195, USA; ^2^Division of Urology, VA Puget Sound Health Care System, 1660 South Columbian Way, S-112-GU, Seattle, WA 98108, USA

## Abstract

With multiple options for urinary diversion after radical cystectomy for bladder cancer that have comparable cancer control and complication rates, health-related quality of life (HRQOL) has become an important consideration. This article reviews the methods for defining HRQOL, the challenges in measuring HRQOL in bladder cancer, and the literature comparing HRQOL after various methods of urinary diversion. Recent contributions include the validation of HRQOL instruments specific to bladder cancer and the publication of several prospective studies measuring HRQOL outcomes after cystectomy and urinary diversion. There is no convincing evidence from existing literature that any particular method of urinary diversion offers superior HRQOL outcomes. Rather, there is growing evidence that good HRQOL can be achieved with patient education and consideration of each patient's clinical and psychosocial situation. Future research should utilize the validated bladder cancer specific HRQOL instruments and perhaps explore the impact of preoperative counseling on postoperative HRQOL.

## 1. Introduction

In 2010 there were estimated 70,530 new cases of bladder cancer in the United States, and over 500,000 current survivors [1, 2]. The standard of care in the United States for muscle-invasive bladder cancer is radical cystoprostatectomy for men and anterior exenteration for women. There is also evidence that earlier cystectomy in high-risk superficial bladder cancer improves long-term survival [3]. Options for urinary diversion after cystectomy include noncontinent conduits, continent cutaneous diversions, and orthotopic bladder substitutes. With the improvement of surgical technique in recent years, continent diversions and orthotopic bladder substitutes have been shown to have similar perioperative complication rates, cancer control, and morbidity [4, 5].

With the proliferation of urinary diversion options for bladder cancer that have comparable cancer control and complication rates, quality of life becomes an important factor to consider. Health-related quality of life (HRQOL) refers to the physical, psychological, and social domains of health that are influenced by a person's experiences, beliefs, expectations, and perceptions [6]. The task of translating the subjective components of health into a quantitative value is a complex one, drawing from a variety of fields in the social sciences. HRQOL is measured with questions, or items, whose answers can be converted to numerical scores. Many questionnaires, or instruments, have been developed to assess the various aspects of HRQOL. An ideal instrument should be valid (measures what it reports to measure), reliable (able to give the same result on several occasions given stable disease), and responsive (able to detect true but clinically meaningful changes). The process of psychometric and clinical testing is beyond the scope of this article, but an instrument can only be correctly considered “validated” if it has undergone the established rigors of developmental testing so that its validity, reliability, and responsiveness can be described quantitatively [7].

 This article reviews the methods for defining HRQOL, the challenges in measuring HRQOL in bladder cancer and the existing literature comparing HRQOL after various methods of urinary diversion.

## 2. Measuring HRQOL

HRQOL instruments can be either generic or disease-specific. Generic instruments are applicable to all patients regardless of their illness. They may address issues like bodily pain, energy or fatigue, and limitations in physical activity that are common to many disease processes. Examples of generic instruments are the Medical Outcomes Study 36-Item Short Form (SF-36) [8] and the Sickness Impact Profile (SIP) [9].

There are multiple instruments that assess cancer-specific HRQOL, including the European Organization for Research and Treatment of Cancer-QOL (EORTC-QLQ-C30) [10] and the Functional Assessment of Cancer Therapy general form (FACT-G) [11]. The current version of the EORTC-QLQ-C30 contains 30 items that are grouped into five functional scales (physical, role, emotional, cognitive, and social), three symptoms scales (fatigue, nausea and vomiting, and pain), and an overall HRQOL scale. The broadness of this instrument makes it generally applicable to all cancer states, but it lacks the specificity to address issues that may be unique to a particular type of cancer [12].

Disease-specific instruments focus on the issues that are relevant to patients with a particular disease state. Some disease-specific instruments are developed from validated generic instruments, with additional items added to make them more specific to the disease of interest. For example, 12 items focusing on urinary tract symptoms, intestinal symptoms, sexual symptoms, and stoma concerns were added to the FACT-G to create the FACT-BL, designed to address HRQOL in bladder cancer. Likewise, 17 items were added to the FACT-G questionnaire to create the Vanderbilt Cystectomy Index (FACT-VCI), which has been separately validated to measure HRQOL following radical cystectomy and urinary diversion for bladder cancer [13]. The European Organization for Research and Treatment of Cancer (EORTC) has also added items to the EORTC-QLQ-C30 core questionnaire to make it specific for superficial and invasive bladder cancer, and is in the process of validating these instruments [14]. More recently, the Bladder Cancer Index (BCI) has been developed and validated to assess health outcomes specific to localized bladder cancer [15]. Refer to Table 1 for a comparison of bladder cancer specific HRQOL instruments.

## 3. Challenges of Measuring HRQOL in Bladder Cancer

 Measuring HRQOL in bladder cancer has its unique difficulties. All of the currently available bladder cancer-specific instruments contain items evaluating the urinary domain, but the items mostly address general problems with urinary control (e.g., “I have trouble controlling my urine” or “I urinate more frequently than usual”). Leaking from a stoma, however, may be a very different experience than leaking per urethra. Different diversions also come with a different set of potential side effects (e.g., metabolic derangements) and impact on body image. It is difficult to account for all these factors in a single questionnaire that can be applied to all patients with bladder cancer.

Consequently, many of the bladder cancer-specific instruments target an even narrower population of bladder cancer patients. As shown in Table 1, the EORTC QLQ-BLS24 is targeted to superficial bladder cancer and will include items related to the bother of repeated cystoscopies, whereas the FACT-VCI was validated to target patients who undergo radical cystectomy for bladder cancer.

In addition, sexual function is an important domain when discussing the impact of bladder cancer and its treatment. Since men and women experience different issues related to sexual function, it is challenging to create a questionnaire that is applicable to both sexes. The BCI sexual domain asks gender-neutral questions addressing sexual desire and arousal, ability to climax, and genital sensation. But men may experience issues with erection and ejaculation, while women may have problems with vaginal lubrication and dyspareunia, which even a disease-specific instrument like the BCI would not be specific enough to distinguish [16].

 On the other hand, as instruments are tailored more specifically to the disease of interest, they may be less likely to detect unanticipated effects of the disease. For example, the BCI contains 36 items distributed among the 3 primary domains that are expected to be relevant in bladder cancer: urinary, bowel, and sexual. If a treatment for localized bladder cancer were to unexpectedly cause a neurologic side effect, this disease-specific instrument would not be sensitive enough to detect this change.

## 4. Current Literature: HRQOL after Cystectomy

In 2005, Porter and Penson published a systematic review examining HRQOL outcomes among different types of urinary diversion after radical cystectomy [17]. After excluding studies that did not specifically address bladder cancer patients, studies that included radiotherapy as treatment, and studies that did not compare at least 2 types of urinary diversion (ileal conduit, orthotopic neobladder, or continent cutaneous urinary reservoir), 15 studies were appropriate for analysis. The review found no randomized, controlled studies. Only 1 study was performed prospectively and included preoperative baseline measurements [18]. The other studies were cross-sectional, consisting of a single-mailed or clinic-administered instrument. Of 15 studies, 10 (67%) used a previously validated health-related quality of life instrument, while 10 (67%) used an instrument developed by the investigators without previous validation, either in conjunction with a validated instrument or as the sole measure of quality of life. The authors concluded that the available data did not conclusively show that any form of urinary diversion was superior to another in terms of HRQOL. 

They also noted that limitations to the literature included the lack of baseline assessment before cystectomy, the lack of longitudinal data to evaluate the impact over time, and the lack of a bladder cancer-specific and validated instrument to measure quality of life. Despite the inability to draw conclusions regarding the superiority of any method of diversion after cystectomy, some common patterns were identified across the studies that may help future research. Three studies indicated that urinary leakage was more of a problem with conduit diversion than with continent diversion [19–21]. Three studies indicated that patients with neobladder or continent reservoir were more likely to travel than patients with conduit diversion [22–24]. Two studies indicated that patients with continent diversions scored better on social function domains than those with conduit diversions [25, 26].

In 2005, Gerharz et al. published a review that rated studies by levels of evidence and grades of recommendations set out by the International Consultation on Urological Diseases modification of the Oxford Centre for Evidence-Based Medicine [27]. They identified no studies with level I evidence and also concluded that there was no evidence to claim that continent reconstruction provides better quality of life than conduit diversion.

Since the publication of these reviews, there have been a handful of studies published in the last 5 years that evaluate HRQOL after cystectomy in bladder cancer. In 2007 Gilbert et al. used the recently validated bladder cancer-specific instrument, the Bladder Cancer Index (BCI), to assess HRQOL in patients treated with a variety of interventions, including cystectomy and endoscopic procedures [28]. They identified all bladder cancer patients in an institutional bladder cancer database from a high-volume tertiary referral center. From this population, 315 (45%) completed the BCI and were included in the study. There was no pretreatment HRQOL assessment. The cases were stratified into 4 groups according to treatment type: native bladder without intravesical treatment, native bladder with intravesical treatment, cystectomy with ileal conduit, and cystectomy with orthotopic neobladder. They found that cystectomy groups scored relatively lower than native bladder groups in all the function and bother domains (urinary, bowel, sexual). Interestingly, the cystectomy with orthotopic neobladder group scored significantly lower in urinary function scores than the other 3 groups, challenging the commonly held belief that continent urinary diversion offers improved HRQOL outcomes compared to incontinent diversion. However, despite the difference in functional scores, the urinary bother scores did not differ between the cystectomy groups, leading the authors to speculate that perhaps neobladder patients are willing to compromise physiologic urinary function in exchange for anatomic urinary function.

Two recent prospective studies looked at body image and its impact of HRQOL. In 2009 Somani et al. published the results of a study that prospectively evaluated 32 patients undergoing radical cystectomy for bladder cancer; 29 ileal conduit diversions and 3 neobladder replacements were performed [29]. The study evaluated HRQOL preoperatively and 9–12 months after cystectomy and urinary diversion using the SEIQoL-DW, a validated generic instrument administered by an independent researcher, and the EORTC QLQ-C30. Patients identified several nonhealth-related determinants of quality of life (family, relationships, finance) that were most important. None of the patients mentioned body image as an important determinant of quality of life. When asked specifically about appearance, 76% in the ileal conduit diversion group said that it was quite or very important but only 14% thought that it would change significantly after the operation. 

In a separate prospective study, Hedgepeth et al. evaluated changes in body image and quality of life in 139 patients undergoing neobladder replacement after cystectomy and 85 patients undergoing ileal conduit diversion, compared to a reference group of patients who had cystoscopy [30]. HRQOL outcomes were measured using the Bladder Cancer Index (BCI), which consists of 36 questions covering urinary, bowel, and sexual health domains. Function is measured by items describing frequency and control of bodily or stoma function, and bother is measured by items describing how much a patient is troubled by their symptoms. Participants were assessed preoperatively, as well as at several time points in the 8 years after surgery. The authors concluded that patients with continent urinary diversions experience greater functional urinary impairment with little difference in overall bother. Body image for both neobladder and ileal conduit diversion patients worsened immediately after surgery but improved in a linear fashion over time. Ileal conduit diversion patients eventually returned to baseline body image levels, with no significant difference from the cystoscopy group in the long-term. Neobladder patients never returned to baseline body image levels.

Acknowledging that the sociocultural milieu in which patients live can influence their perception of health and illness, studies have also begun to explore the role of cultural differences in HRQOL. In a 2007 prospective study comparing male patients from Sweden and Egypt undergoing radical cystectomy and orthotopic neobladder substitution for locally advanced bladder cancer, Månsson et al. reported that Egyptian men were more likely to express depression and anxiety on the Hospital Anxiety and Depression Scale (HADS), a validated generic instrument to detect mood disorders, as well as score lower on the FACT-BL than their Swedish counterparts [31]. The authors did warn against pitfalls in comparing patients from different cultural groups, such as the inherent demographic differences that may bias the results, as well as the difficulty of preserving conceptual and linguistic equivalence when translating HRQOL questions into a different language. However, this represents an important step in acknowledging that patient-assessed outcomes differ in patients from different sociocultural backgrounds.

Investigators are also beginning to evaluate HRQOL in patients undergoing bladder preservation therapy in the setting of invasive bladder cancer. Lagrange et al. in a prospective, multicenter study published this year evaluated 53 patients with muscle-invasive disease in a Phase II trial who underwent pelvic radiation and concurrent chemotherapy with cisplatin and 5-fluorouracil [32]. Subjects completed the EORTC QLQ-C30 pretreatment, as well as at several time points post treatment. The results indicated that 70% maintained good scores for bladder function for 24 months, at which point deterioration in symptoms, such as frequency, pain, and urinary control, was seen. This study is limited by the lack of a disease-specific instrument, and there are not yet any studies comparing HRQOL in bladder preservation therapy with the standard of care, cystectomy with urinary diversion.

## 5. Conclusion

Improvements have been made in recent years in the field of HRQOL research in bladder cancer. More prospective studies are being published with HRQOL instruments administered prior to treatment as well as at multiple points in time after treatment. However, the vast majority of available studies are still retrospective and cross-sectional. This study design is less able to distinguish differences in health status that may simply reflect underlying preoperative differences.

Another major limitation to existing literature is the lack of an available bladder cancer-specific, validated instrument to measure health-related quality of life. With the development of the Bladder Cancer Index, as well as the growing popularity of the FACT-BL and the bladder cancer module of the EORTC QLQ-C30, researchers should be better equipped to assess HRQOL in bladder cancer patients.

Even with the improvements of the last 5 years, there is still not conclusive evidence that any type of urinary diversion offers superior HRQOL outcomes. In fact, though postcystectomy patients commonly experience urinary and sexual problems, HRQOL remains good irrespective of the method of urinary diversion. It has been speculated that the reason for this finding is that patients who receive thorough preoperative counseling end up choosing the method that is most suitable for them and are well prepared for the adjustment after surgery [27]. It seems that patient education, careful evaluation of each patient's unique clinical and psychosocial situation, and active patient participation in treatment decisions remain crucial to good postoperative quality of life. 

Although progress has been made in evaluating HRQOL in postcystectomy bladder cancer patients, there is still a need for well-designed, prospective studies. In light of recent evidence demonstrating that despite marked differences in functional outcomes, body image and ultimately overall bother are similar between methods of urinary diversion, it would be interesting to conduct studies looking at the way preoperative counseling and patient education impact HRQOL post treatment.

## Figures and Tables

**Table 1:** Comparison of bladder cancer specific HRQOL instruments.

	*Target population*	*No of items *	*Domains*				*Validation*
			General well-being	Bowel (no. of items)	Urinary (no. of items)	Sexual (no. of items)	
Bladder Cancer Index (BCI)	Localized bladder cancer	36	No	Yes (10)	Yes (14)	Yes (12)	Test-retest 0.73–0.95, internal consistency 0.77–0.91
EORTC QLQ-BLS24	Superficial bladder cancer (Ta, T1, CIS)	24	No	Yes	Yes (including items related to intravesical treatment: fever, malaise, bother of repeated cystoscopies)	Yes	Pending
EORTC QLQ-BLM30	Invasive bladder cancer (T2, T3, T4a/b)	30	No	Yes	Yes (including items related to urostomy probems, catheterization)	Yes	Pending
FACT-BL	Bladder cancer	45	FACT-G	Yes (4)	Yes (3) + Stoma (2)	Yes (2)	FACT-G: test-retest 0.82–0.92
FACT-VCI	Bladder cancer, after radical cystectomy	45	FACT-G	Yes (4)	Yes (4)	Yes (4)	Test-retest 0.79, internal consistency 0.83–0.85
